# Newcastle disease vaccine virus I-2 fails to acquire virulence during repeated passage *in vivo*

**DOI:** 10.12688/gatesopenres.13212.1

**Published:** 2021-04-19

**Authors:** Shahn P.R. Bisschop, Andrew Peters, Gil Domingue, Michael C. Pearce, Jeanette Verwey, Petrus Poolman

**Affiliations:** 1 Avimune (Pty.) Ltd, Queenswood, Pretoria, South Africa; 2 Poultry Reference Centre, Department of Production Animal Studies, Faculty of Veterinary Science, University of Pretoria, Pretoria, South Africa; 3 GALVmed, Pentlands Science Park, Midlothian, Scotland, UK; 4 Supporting Evidence based Interventions-Livestock, University of Edinburgh, Easter Bush Campus, Midlothian, Scotland, UK; 5 GD Associates, Fairmilehead, Edinburgh, Scotland, UK; 6 Epidemiological Research Unit, Scotland’s Rural College, An Lòchran, Inverness, UK; 7 Deltamune, Roodeplaat, Pretoria, South Africa; 8 Kuipers Group (Pty.) Ltd, Zeekoeigat, Pretoria, South Africa

**Keywords:** Newcastle Disease, I-2; passage, acquired virulence, European Pharmacopoeia, village chickens

## Abstract

**Background** This study determined whether the naturally attenuated, thermotolerant Newcastle disease vaccine virus I-2 could acquire virulence after five *in vivo* passages through SPF chickens.

**Methods** Study design was to international requirements including European Pharmacopoeia, Ph. Eur., v9.0 04/2013:0450, 2013. I-2 Working Seed (WS) was compared with five-times-passaged I-2 WS (5XP WS) in intracerebral pathogenicity index (ICPI), F _o_ cleavage site sequencing and Safety tests.

**Results** The first passage series used a 50% brain: 50% tracheal tissue challenge homogenate and was unsuccessful as I-2 was not detected after the fourth passage. A second passage series used 10% brain: 90% tracheal tissue homogenates. I-2 was isolated from tracheal tissue in each passage. However harvested titres were below the minimum challenge level (10 ^7^ EID _50_) specified for the ICPI and Safety tests, possibly reflecting I-2’s inherently low pathogenicity (interestingly caecal tonsils yielded significant titres). Given this the WS and 5XP WS comparisons proceeded. ICPI values were 0.104 and 0.073 for the WS group and the 5XP WS group respectively confirming that I-2, whether passaged or not, expressed low pathogenicity. F _0 _amino-acid sequences for both WS and 5XP WS were identified as ^112^R-K-Q-G-R-↓-L-I-G ^119^ and so compatible with those of avirulent ND viruses. In safety, no abnormal clinical signs were observed in both groups except for two chicks in the 5XP WS group, where one bird was withdrawn due to a vent prolapse, and another bird died with inconclusive necropsy results.

**Conclusions:** These data, the issue of low passage titres with little or no virus isolation from brain tissues and the genomic copy approach suggest a need to amend Ph. Eur. v9.0 04/2013:0450, 2013 for naturally attenuated, low pathogenicity vaccine viruses such as I-2. These results add to the literature and field data demonstrating that Newcastle Disease vaccine virus I-2 is safe for use.

## Introduction

Newcastle disease (ND) is one of the most important viral diseases of poultry and occurs in both commercial flocks and also in scavenging rural (village, backyard, sector 4,) chickens ( Alders & Spradbrow, 2001; Cattoli *et al.,* 2011; FAO, 2007; Spradbrow, 2000). The latter birds contribute significantly to the economies of poor households by providing eggs and meat for consumption and sale or bartering. These birds also are sources of readily available cash and gifts and may also have ceremonial or ritual value ( Moreki *et al*., 2011; Perry *et al.,* 2002; Peters *et al.,* 2012a and Peters *et al.,* 2012b). They require the lowest capital investment of any livestock species, and have a short production cycle ( Copland & Alders, 2004). Unfortunately the behaviour of village birds is suited to disease spread, as despite being from multiple households, chickens will often congregate when scavenging so in effect forming one large village flock of all-ages and with unknown genetics. This enhances the transmission of infectious agents which has major implications for vaccination strategies ( Msoffe *et al.,* 2010). ND epidemics can result in up to 100% morbidity and 100% mortality in unvaccinated flocks with disastrous socio-economic consequences for both commercial and small poultry keepers ( Alexander, 2000; Alexander & Senne, 2008; GALVmed-PANVAC-IIAM, 2009; Spradbrow, 2000) so potential losses due to ND make vaccination mandatory ( Copland & Alders, 2004). 

Currently only one serotype of ND virus is recognised and control of ND by vaccination is widely practised in commercial poultry flocks. However the vaccines used in the commercial sector are less suited for use in the village chickens of low–middle income countries ( Aini *et al*., 1990; GALVmed-PANVAC-IIAM, 2009). Typically, commercial vaccines are produced in large dose vials, are often insufficiently thermotolerant so require a dependable cold-chain and generally are too expensive for use in village flocks. These issues led to the characterisation of thermotolerant ND virus vaccine strains that could be produced inexpensively in smaller batches by local laboratories for use in all-age backyard flocks ( Campbell *et al.,* 2019; Domingue *et al.,* 2017; Spradbrow 1993, 1994). An example of such a vaccine is the naturally attenuated, thermotolerant I _2_ ND virus, now commonly known as I-2, which has long been known to be a suitable vaccine for use in developing countries. This is due partly to its high titre yield in embryonated eggs, its lack of virulence, a low pathogenicity coupled to a high immunogenicity which confers substantial protection, its thermotolerance (at least 12 weeks when stored at 22 ^o^C in 1% gelatin), its straightforward delivery routes including by eye drop, its contact spread between birds and its safety and efficacy in very young (8 day) African local ecotype chicks ( ACIAR, 2005; Bensink & Spradbrow, 1999; Copland & Alders, 2004; Dias *et al.,* 2001; Domingue *et al.,* 2017; Henning *et al.,* 2009; Kattenbelt *et al.,* 2006; Tu *et al.,* 1998; Wambura *et al.,* 2000; Wambura *et al.,* 2006; Wambura *et al.,* 2007). While the ND I-2 vaccine is thermotolerant, it eventually loses its potency if exposed to excessive sunlight or temperatures for long periods, i.e. it is not thermostable ( Copland & Alders 2004). This was partly addressed for thermotolerant ND vaccines in general by Domingue *et al.,* 2017 who demonstrated that a preparation of 10X field dose of Clone 30 could offset viability loss due to high temperature (24 h, 32.3 ^°^C) while retaining safety and efficacy.

I-2 Master Seed (MS) is maintained by the University of Queensland, Australia and owned by the Australian Centre for International Agricultural Research (ACIAR) who make it available at no cost to low-middle income countries wishing to establish local ND vaccine production. ( Alders & Spradbrow, 2001). ND I-2 vaccine use has also been allowed in these low-middle income countries because local registration requirements are relatively relaxed, but the vaccine has not been registered in those countries where registration requirements are more demanding.

The Global Alliance for Livestock Veterinary Medicines (GALVmed) is a not-for-profit organisation that helps develop and register veterinary medicines for livestock in those markets that are not attractive to the global commercial animal health industry. GALVmed has prioritised livestock diseases in low - middle income countries depending on perceived unmet need irrespective of species and target diseases, and so has included ND in poultry for development funding.

Despite I-2’s long known suitability, there is a need to develop a globally acceptable I-2 registration dossier, especially to help AU-PANVAC in its pro-poor aims, as agreed with the Bill and Melinda Gates Foundation. Indeed one of the obstacles to the wider use of the I-2 vaccine has been that no comprehensive dossier has been compiled on the vaccine. This has delayed or prevented registration of the product in a number of low-middle-income countries. Data confirming that the vaccine virus does not increase in virulence after serial passage through chickens is an essential component of such a dossier.

Therefore there was a universal requirement to re-investigate the naturally attenuated I-2 ND vaccine strain but under appropriate regulations to determine whether it would *acquire* virulence after five serial *in vivo* passages in SPF chickens. Accordingly, GALVmed sponsored a trial to good laboratory principles (GLP) ( OECD, 1998) at the Veterinary Faculty of the University of Pretoria at Onderstepoort, South Africa. The study design incorporated the demands of the European Pharmacopoeia (Ph. Eur.), 2013; VICH GL 41, 2007 and the European Union Council Directive 81/852/EEC, 1981 to meet the requirements of a large number of countries so that the vaccine might be widely accepted by international registration authorities.

## Methods

### I-2 Newcastle disease (ND) virus

Master seed (MS) was a gift from ACIAR (Australian Centre for International Agricultural Research). Preparation of MS and working seed (WS) was as described ( ACIAR, 2005). Briefly MS was reconstituted, titrated, diluted and inoculated into embryonated eggs. After 4 days incubation the allantoic fluids were pooled and the WS harvest was titrated, adjusted to 4.0 × 10 ^9^ EID _50_ ml ^-1^/ 8.4 × 10 ^10^ GC, genomic copies, ml ^-1^ and stored at -70 ^o^C.

### Study practice and design

**Figure 1.**
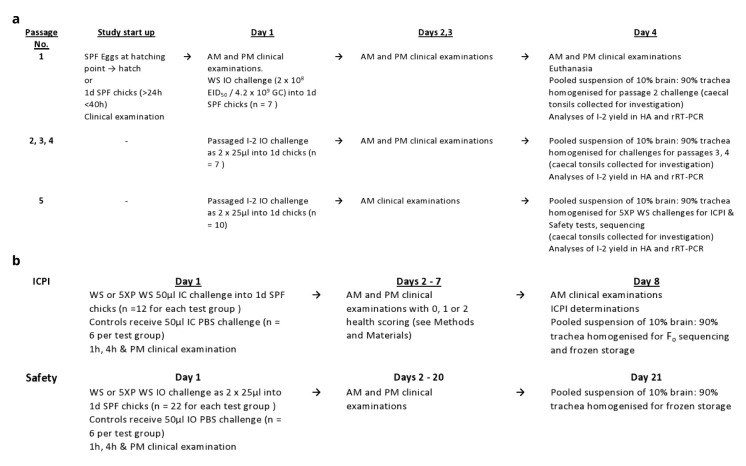
Flow design for ( **a**) second series 5 times *in vivo* passaging of I-2; ( **b**) ICPI, F _o_ sequencing and Safety tests of I-2.

Design complied with international regulatory requirements ( Ph. Eur. v9.0 04/2013:0450, 2013; VICH GL41, 2007; EU Council Directive 81/852/EEC, 1981) to ensure global acceptance. Study practice including data collection and storage was to Good Laboratory Practice (GLP) -like principles ( OECD, 1998). All Study practice and design activities were performed under the umbrella of the GALVmed Quality Control system ( VICH GL41, 2007 standard). The study flow designs are shown in Figure 1a and 1b.

Briefly, the prescribed test for “increase in virulence” ( European Pharmacopeia, Ph. Eur. v9.0 04/2013:0450, 2013) requires that native I-2 ND vaccine virus be administered to birds at “the least attenuated passage level present between the MS lot and a batch of vaccine”. Since only one vial of ND I-2 MS was available, the least attenuated passage level was confirmed as Working Seed (WS; Ph. Eur. Helpdesk, personal communication).

The test for increased virulence, i.e. *acquisition* of virulence in the case of the naturally attenuated I-2, starts with the 5 times *in vivo* passage with at least 5 chicks per passage, of I-2 WS (i.e. native or non-passaged) to produce 5XP WS (i.e. 5X passaged WS).

The prescribed test then continues as the detection of any virulence changes in ICPI (Intracerebral pathogenicity index), F _o_ amino acid sequence and Safety assessments (see below and Figures 1a and 1b).

### Five-times *in vivo* passage

Ph. Eur. v9.0 04/2013:0450, 2013 requires the administration by the intra-ocular (IO) route, of a quantity of I-2 virus in homogenates containing both brain and tracheal tissues, that will allow recovery of I-2 virus through the 5 passages.

There were two passage series and each used seven SPF 1-day-old chicks per passage. In both series, to attempt the required I-2 recovery, the initial IO challenge, i.e. the challenge into the first passage birds, was 2.0 × 10 ^8^ EID _50_/ 4.2 × 10 ^9^ GC via 2 x 25 µl drops of native I-2 WS at 4 × 10 ^9^ EID _50_ ml ^-1^/ 8.4 × 10 ^10^ GC ml ^-1^. Next, for both series, four sequential IO challenges i.e. numbers 2 to 5 (2 x 25µl eye drops of pooled organ homogenate from the previous passage) and passages followed. Caecal tonsils were also collected in both series and investigated for viral titres, as although not demanded by the Ph. Eur. v9.0 04/2013:0450, 2013, this was normal procedure at the study site; N.B. caecal tonsils were never incorporated into any homogenates.

Critically, the two series differed in their homogenate compositions. The first passage series was for “range-finding” and used an initial IO challenge homogenate made up of 50% brain: 50% tracheal tissue. During each passage, birds were observed twice daily for 4 days, subsequently euthanised (cervical dislocation) and a pooled suspension of homogenised brain and tracheal tissue was prepared for the next passage. The presence of I-2 virus in the pooled suspension of homogenised brain and tracheal tissue from each bird in each passage was detected by egg passage and subsequent EID _50_ in HA and by real-time reverse transcription-polymerase chain reaction (rRT-PCR) (see below). Unfortunately, this first passage series was incomplete as I-2 virus could not be detected after the fourth passage and throughout I-2 virus yields from brain tissue were very low or negative (see *Results*).

In accordance with the Ph. Eur. v9.0 04/2013:0450, 2013 and implementing advice from the Ph. Eur. Helpdesk, the second passage series was performed. An “augmented” homogenate consisting of 10% brain: 90% tracheal tissue to minimise the dilution effect of the brain material, was used as the challenge inoculum for passages 2 to 5. This approach was successful in that I-2 was isolated to the end of the fifth passage, albeit in very low titres. Although these were below the minimum challenge level of >2.0 × 10 ^8^ EID _50 _ml ^-1^ (i.e. 50 µl containing10 ^7^ EID _50_) specified by the Ph. Eur. v9.0 04/2013:0450, 2013 for the challenges for the subsequent ICPI and Safety tests, an exploratory approach was taken and the five times passaged I-2 was assessed against native WS I-2 in the ICPI, amino acid sequencing of the F _0_ cleavage site and Safety tests as described below.

A.    ICPI: a daily scoring of signs for 8 days after intracerebral (IC) administration (0 = normal, 1 = clinical signs of disease, 2 = dead). For a virus challenge of not less than 10 ^8^ EID _50_ or for a virus challenge less than 10 ^8^ EID _50_ but not less than 10 ^7^ EID _50_, a virus would comply with the test if the ICPI induced was not greater than 0.5 or 0.4, respectively.

B.    Determination of the encoded amino acids at the I-2 F _0_ cleavage site that imparts pathogenicity in ND viruses.

C.    Safety, an absence of clinical signs after ocular challenge, IO, the recommended vaccination route for I-2.

### Bird numbers

To allow for mortality and to ensure that no treatment group included fewer birds than required in the Ph. Eur. monograph above, the treatment group sizes were increased slightly above monograph requirements per group:

Passage - 7 birds per passage for the first four passages and 10 birds for the fifth passage
ICPI - 12 birds
Safety - 22 birds

Groups of 6 birds each were present as negative controls for each test that included virus passages and safety tests on the WS and 5XP WS. Further groups of 6 chicks served as controls for the ICPI techniques.

### Location

The study was conducted at the National Department of Agriculture-approved BSL3 laboratory isolation unit at the University of Pretoria, South Africa.

### Ethics

Approval for the study was obtained in advance from the University of Pretoria Animal Use and Care Committee as well as the Research Committee of the Veterinary Faculty. Approval for the study was also obtained in advance from the National Department of Agriculture. These approvals and the GALVmed Quality Control System ensured that the study followed the principles of the Animal Research: Reporting of In Vivo Experiments (the ARRIVE full set 2.0) guidelines ( Kilkenny *et al.,* 2010). Any trial amendments were also approved by the above committees of the University of Pretoria.

### Chickens

Mixed sex, specific pathogen free (SPF) Leghorn chickens were obtained either as eggs on the point of hatching or as day-old chicks (1 day; >24 h and <40 h; Deltamune (Pty) Ltd, SA). Birds (n = 290 in total) were placed immediately after hatching into approved BSL3 isolation cabinets (12 per cabinet; different treatment groups were allocated to separate isolation cabinets; Horsefall isolation units, SA) and remained housed within the BSL3 unit until euthanasia.

### Inclusion/exclusion/withdrawal criteria for study chickens

***Inclusion criteria***

- Day-old chicks (chickens) hatched from SPF eggs.
- Chicks were clinically normal and in good health.

***Exclusion criteria***

- Unhealthy chicks
- In the event that more healthy chicks hatched than were required for the study, the study director excluded the smallest birds from the study.

***Withdrawal criteria***

- Any abnormal signs considered to compromise the welfare of the chicken.
- Any chicken requiring concurrent medication that could compromise its suitability for the study.

***Randomisation and bias reduction*.** Randomisation plans were prepared by an independent biometrician. Chicks were individually identified using food colouring and/or coloured leg bands, so they could be assigned numbers and randomly allocated to treatment groups. Chicks were randomised to treatment and randomised between isolation cabinets. For some passages, no randomisation was performed as each treatment group was placed sequentially and on different days. For all five passages, chicks in each isolator were individually marked using coloured leg bands. This was solely for the purpose of monitoring, so that investigators were able to determine if an individual chick had been in protracted recumbency or had consistently exhibited particular clinical signs. Post-challenge observation and clinical scoring was conducted by two observers and by different observers throughout the different phases of the study in order to reduce observer bias.

### Five-time *in vivo* passage (5XP) of I-2 WS and comparisons of 5XP I-2 with native I-2 WS

***Tissue homogenisation (for preparation of passage challenges)*.** Post-mortem, organs were placed into a solution at a dilution of 1 part tissue: 4 parts PBS (phosphate buffered saline, pH7.4) containing antibiotics (penicillin 1,000 units ml ^-1 ^and streptomycin 10,000 μg ml ^-1^) and an anti-mycotic (amphotericin B 25 μg ml ^-1^) and blended.

***I-2 quantification*.** All challenges for the *in vivo* passaging, ICPI and safety tests were quantified at time of use in the HA and rRT-PCR assays below as were all test yields.

***Quantification by haemagglutination inhibition (HAI) test*.** After each *in vivo* passage in chickens, organ homogenate was inoculated into the allantoic cavity of a series of 11-day embryonated SPF eggs and the EID _50_ calculated according to the method of Reed & Muench (1938) using the macroscopic haemagglutination technique ( Anon, 1989).

***Quantification by rRT-PCR*.** This was implemented for I-2 virus quantification because post-passage yields were very low making quantification by the gold standard of HA unreliable or impossible. The authors acknowledge that the Ph. Eur. v9.0 04/2013:0450, 2013 monograph does not consider genomic copy units.

***RNA extraction*.** I-2 RNA was extracted from homogenised tissue samples or allantoic fluids using Tri-Reagent (TR 118, Molecular Research Center Inc.; Cincinnati, OH, USA) followed by further purification of RNA using the Qiagen RNeasy® MinElute™ Cleanup Kit (Cat No 74204; Qiagen, The Scientific Group, Gauteng, South Africa) and elution of RNA in 15 µl of RNase-free water (Cat No 129112; Qiagen). Three µl of RNA was used in RT-PCR reactions with final volumes of 10 µl.

***Primers and probes*.** The primers NDF and NDR were used in combination with probe “NDpro2” as described in Fuller *et al*., 2010. Primer and probe sequences were confirmed prior to testing and the probe sequence was confirmed as identical to the published sequence of ND I-2 virus. Primers and Probes were synthesised by TibMolbiol, Berlin, Germany. Primers were used at a final concentration of 200 µmolar and the probe at a final concentration of 100 µmolar in each reaction.

***Assay details*.** The rRT-PCR of Fuller *et al.,* 2010 was adapted ( Jang *et al.,* 2011) to a Roche LightCycler® 480 and dedicated reagents (Hoffmann-La Roche Ltd, South Africa). After optimisation, the LightCycler® RNA Amplification Kit HybProbe (Cat No 12015145001; Hoffmann La Roche) with a final MgCl _2_ concentration of 7 mM was used. A total of 3 µl purified RNA was used to give a final 10 µl reaction volume.

Cycling parameters were as follows: 55°C for 10 minutes – (reverse transcription), 95°C for 1 minute – (initial denaturation); followed by 40 cycles of amplification followed by 95°C for 5 seconds (denaturation), 55°C for 5 seconds (primer binding), 60°C for 20 seconds (hydrolysis of probe and acquisition of fluorescence); cooling to 40°C for 10 seconds.

In each run, in order to quantify virus, at least three standard solutions containing known amounts of Newcastle Disease virus I-2 GC were also analysed – the standards were prepared after purification of ND I-2 on a sucrose gradient, extraction of RNA from purified virus, and quantification of RNA (8.1 ng of NDV RNA represents 10 ^9^ NDV GC. From a stock containing 10 ^9^ GC μl ^-1^, 10-fold serial dilutions were made from 10 ^9^ to 1.0 GC μl ^-1^. Quantification of RNA copies per ml of homogenised sample were calculated by multiplication of the genomic copies per PCR reaction by the dilution factor used during RNA purification.

### Assessment of possible increase in virulence

***ICPI test*.** Each bird in the WS group (n=12) was given an intracerebral (IC) challenge of 50 μl from pooled allantoic stock containing 4 × 10 ^9^ EID _50_ ml ^-1^/ 8.4 × 10 ^10^ GC ml ^-1^ of I-2 ND virus (i.e. 2 × 10 ^8^ EID _50_ / 4.2 × 10 ^9^ GC total).

Those birds (n=12) challenged with 5x passaged WS received 50 μl IC of a homogenate composed of 90% tracheal tissue containing 5.1 × 10 ^6^ EID _50_ ml ^-1^/ 4.1 × 10 ^7^ GC ml ^-1^ of I-2 and 10% brain tissue with no I-2 detected in both rRT-PCR and HAI. The total I-2 challenge was 2.6 × 10 ^5^ EID _50_/ 2.1 × 10 ^6^ GC which was below the minimum challenge level ( Ph. Eur. v9.0 04/2013:0450, 2013) for the ICPI tests. 

Two control groups (n=6 each), one for each test group above, received a 50 μl IC challenge of PBS intracerebrally. Test and control birds were observed (clinical administration post vaccine administration, CEPVA) at 1 h and 4 h post-treatment and then at least twice daily for 8 days.

An ICPI was calculated for the chickens according to the Ph. Eur. v9.0 04/2013:0450, 2013. The ICPI is the mean of the scores per bird per CEPVA, performed twice a day, over an 8-day period: 0 = clinically normal; 1 = clinical signs of disease; 2 = dead. I-2 would comply with the test if the ICPI induced was not greater than 0.5 or 0.4 respectively.

***F _o_ amino acid sequencing*.** Pooled, homogenised 10% brain: 90% trachea samples were analysed. The encoded amino acids at the F _0_ cleavage site in RNA preparations from I-2 WS and 5 XP ND I-2 were determined via the RNA sequencing service of the Molecular Epidemiological and Diagnostic Programme, Agricultural Research Council - Onderstepoort Veterinary Institute, Pretoria, South Africa. Sequencing reactions were prepared using a BigDye Terminator v3.1 sequencing kit (Applied Biosystems, USA) and a 3500/3500xL Genetic Analyzer (Applied Biosystems). Multiple sequence alignments were performed using the BioEdit v 7.1 sequence alignment ( Hall, 1999). The derived amino acid sequences were obtained using the ExPASytranslation tool (v 2003; Gasteiger *et al.,* 2003).

***Safety test*.** Each bird in the native WS group (n=22) received 50 μl of 4 × 10 ^9^ EID _50_ ml ^-1^/ 8.4 × 10 ^10^ GC ml ^-1^ of I-2 administered IO as two 25 μl drops (2 × 10 ^8^ EID _50_ / 4.2 × 10 ^9^ GC total challenge).

 Each bird in the 5XP WS group (n=22) received 50 μl of a homogenate composed of 10% brain tissue (I-2 not detected in rRT-PCR and HAI): 90% tracheal tissue (containing 5.1 × 10 ^6^ EID _50_ ml ^-1^/ 4.1 × 10 ^7^ GC ml ^-1^ I-2) and administered IO as two 25 μl drops (2.6 × 10 ^5^ EID _50_/ 2.1 × 10 ^6^ GC total challenge). Again this was below the minimum challenge level specified by the Ph. Eur. v9.0 04/2013:0450, 2013.

Negative control birds (n=6) for each safety test received PBS pH 7.4 in two 25 μl eye drops. All birds were observed at 1h and 4h post-treatment and then at least twice daily for 21 days.

***Control birds*.** All controls were examined for I-2 virus as above to detect possible accidental cross-inoculation except that for the rRT-PCR, choanal cleft swabs were pooled and analysed. Also “sentinel” chickens were placed to monitor biosecurity throughout the study.

## Results

### Control birds

Negative, untreated and placebo control groups were present in the same study rooms during the I-2 *in vivo* passages, the safety tests and the ICPI tests. No abnormal clinical signs were observed in any negative control chickens. ND I-2 was never detected in any tissue samples. Again I-2 was never isolated from “sentinel” chickens indicating that biosecurity was satisfactory throughout the study.

***In vivo passages*.** Only one bird was withdrawn from the study. This bird subsequently died from a cloacal prolapse which was judged not to be linked to the challenge. No birds died in any of the *in vivo* passage groups, or in the control groups.

No signs of clinical disease in chickens were observed as a result of challenge with ND I-2 WS, whether native or five-times-passaged material (organ homogenate), in any of the passages. The first attempt at passaging was not successful in that virus could not be detected after the fourth passage but it was noted that viral loads in the tracheal tissues were 3–5 times greater than in the brain tissues (data not shown).

In the second series of passages, an augmented homogenate consisting of 10% brain: 90% tracheal tissue was used for the IO challenges for each group and this proved more successful as 5XP I-2 WS was obtained. The HA and rRT-PCR analyses of the tissues recovered from each passage are shown in Table 1. Significantly, I-2 was not detected in brain tissues through all passages and overall, virus titres in all harvested tissues were below the minimum challenge level of >2.0 × 10 ^8^ EID _50 _ml ^-1^ (i.e. 10 ^7^ EID _50_ in 50 µl per chick) specified in the Ph. Eur. v9.0 04/2013:0450, 2013 for the challenge for the ICPI and Safety tests: the highest tracheal yields for I-2 were 5.1 × 10 ^6^ EID _50_ ml ^-1^/ 4.1 × 10 ^7^ GC ml ^-1^ (passage 5) while those for caecal tonsils were 1.8 × 10 ^7^ EID _50_ ml ^-1^/ 6.0 × 10 ^8^ GC ml ^-1^ (passage 2). Despite the low HA titres, it was decided that, given the exploratory nature of this work, to continue with the ICPI and Safety tests with a challenge homogenate composed of both brain and tracheal tissue to ensure compliance but with the former at only at 10%, to minimise the dilution effect of the brain material.

**Table 1.** Second series of five *in vivo* passages: I-2 virus quantification in harvested tissues *.

	Passage Number											
	1		2		3		4		5		Mean ± SEM	
**Tissue**	EID _50_ ml ^-1^ ^a^	GC ml ^-1^ ^b^	EID _50_ ml ^-1^	GC ml ^-1^	EID _50_ ml ^-1^	GC ml ^-1^	EID _50_ ml ^-1^	GC ml ^-1^	EID _50_ ml ^-1^	GC ml ^-1^	**EID _50_ ml ^-1^**	**GC ml ^-1^**
Brain	ND ^c^	ND	ND	ND	Possible ’unreliable positive’ ^d^	ND	ND	Not done	ND	ND	-	-
Trachea	1.0 × 10 ^6^	6.0 × 10 ^8^	1.0 × 10 ^4^	6.2 × 10 ^8^	4.3 × 10 ^6^	1.1 × 10 ^8^	4.0 × 10 ^6^	3.6 × 10 ^7^	5.1 × 10 ^6^	4.1 × 10 ^7^	2.27 ± 1.93 × 10 ^7^	2.81 ± 1.35 × 10 ^8^
Caecal Tonsils ^e^	1.0 × 10 ^5^	5.0 × 10 ^5^	1.8 × 10 ^7^	6.0 × 10 ^6^	1.3 × 10 ^6^	1.4 × 10 ^7^	1.4 × 10 ^4^	6.5 × 10 ^6^	1.6 × 10 ^6^	9.1 × 10 ^6^	4.2 ± 3.46 × 10 ^6^	7.22 ± 2.2 × 10 ^6^

* after initial IO challenge (2 x 25µl drops of native I-2 WS containing 2 × 10 ^8^ EID _50_ / 4.2 × 10 ^9^ GC) into first passage birds and 4 subsequent IO challenges (challenge titres are given in table) **a** Haemagglutination Inhibition test **b** rRT-PCR test **c** ND, I-2 not detected **d** unreliable as positive and negative results were obtained on alternate testing and re-testing; this may have been because despite all precautions, uneven distribution of very low numbers of I-2 particles occurred due to aggregation. **e** caecal tonsils sampling not required by Ph. Eur. v9.0 04/2013:0450, 2013; collected as part of normal site practice.

### Comparative tests

***ICPI*.** Clinical observations were twice daily. For WS, 4 of 12 birds (33.3%) showed clinical signs as a result of ill-health and an unwillingness to drink associated with the effects of the ND challenge as observed post-mortem. Two other birds were also seriously ill by the end of the study. There were two deaths due to dehydration shortly before the end of the study in this group - one bird was sick on day 7 and was found dead on the morning of day 8. One bird was noted as sick on the morning of day 8 and died on the final morning, day 9. Additionally, one bird was recorded as being sick on days 8 and 9 while a final bird was found sick on the morning of day 9. The ICPI value for the native ND I-2 WS was 0.104 for 96 possible observations i.e. 10/ 96 = 0.104. The native WS I-2 therefore complied with the test with an ICPI not greater than the cut out value of 0.4.

For 5XP WS, two of 12 birds (16.7%) showed depression and died shortly before the end of the study on day 8 of observation. On post-mortem examination, both birds showed signs of dehydration. Two (16.7%) other birds were also seriously ill by the end of the study and three birds showed decreased activity when compared with birds in the ICPI control group. The ICPI value for the 5XP I-2 WS was 0.073 for 96 possible observations i.e. 7/ 96 = 0.073. The 5XP I-2 virus seemingly complied with the test as its ICPI was less than 0.4 but this is qualified as the challenge used was less than that required by the Ph. Eur. v9.0 04/2013:0450, 2013.

For both test groups, the negative control chickens showed no abnormal clinical signs related to the IC PBS challenge.

***Amino acid sequencing*.** Samples from WS and 5XP WS were identified by BLAST analysis of the cDNA sequence as ND I-2 vaccine strains. The translated amino acid sequences at the fusion protein cleavage site (F _O_) for both WS and 5XP WS were identical, namely ^112^R-K-Q-G-R-↓-L-I-G ^119^ and further, were consistent with those of avirulent ND viruses,

***Safety*.** Generally no clinical signs of abnormal health were seen in chickens from both native WS I-2 and 5XP WS I-2, except for two chickens in the 5XP WS group: one bird developed a vent prolapse and was consequently excluded from the study, while another bird showed neurological signs and lateral recumbency before death. The post-mortem for this bird was inconclusive but dehydration signs were observed. Both native WS I-2 and 5XP WS 1-2 (given the low challenge level) had complied with the test as the cut out of < 10% of birds dying was not exceeded. The control birds never showed abnormal clinical signs.

## Discussion

The Ph. Eur. v9.0 04/2013:0450, 2013 requires that all tissue homogenate challenges for *in vivo* passage must contain brain tissue. The first five times passage series used a 50% brain: 50% trachea ratio, meaning that I-2 in tracheal tissue was diluted by brain tissue (I-2 not detected) rendering the first series invalid. The second passage series used a homogenate ratio of 10% brain: 90% trachea which was more effective but again I-2 was not detected in brain tissue. The titres in tracheal tissues were highest after the fifth passage at 5.1 × 10 ^6^ EID _50_ ml ^-1^ but still below the minimum challenge level of >2.0 × 10 ^8^ EID _50 _ml ^-1^ required for the ICPI and Safety tests ( Ph. Eur. v9.0 04/2013:0450, 2013). This seemed to indicate that the birds were holding the infection at low levels throughout the passaging, which probably reflected the inherently low level of pathogenicity for ND I-2 (Dr Peter Spradbrow, personal communication). Significantly, no evidence was obtained of “conditioning” or habituation of the I-2 virus that would have resulted in increasing yields through the passages, for both tracheal and caecal tonsils tissues. The Ph. Eur. v9.0 04/2013:0450, 2013 notes that if virus (i.e. I-2) is not recovered after the two passage series, then it is compliant with the test. Despite the low 5XP WS I-2 titres, it was decided for exploratory reasons to use the available material and continue with the ICPI, sequencing and safety tests.

In the ICPI tests, some birds died in both WS and 5XP WS groups subsequent to IC. This occurred towards the end of the trial period. The mortalities were in a small number of birds such that the ICPI values calculated were well within acceptable limits ( Ph. Eur. v9.0 04/2013:0450, 2013) and low when compared to results for control birds. 5XP WS did not acquire virulence as partial genomic sequencing showed no changes in the structure of the fusion protein site F _o_. In the safety tests, one bird in the 5XP WS group died despite the low titre challenge, from unknown causes that could not be distinguished from the possible effects of ocular challenge with ND I-2 virus. According to the Ph. Eur. v9.0 04/2013:0450, 2013, this single death rendered the safety test inconclusive. However, the five-times-passage data, the ICPI and rRT-PCR sequencing data and the apparent lack of conditioning contrasted strongly with the Safety results. The sequencing data for WS and 5XP WS confirmed no change within the pathogenicity locus. However other sequences were not examined so it is possible that other pathogenicity sequences in 5XP WS were modified (upregulated) by the *in vivo* passages to give this deleterious effect (the I-2 genome with Genbank accession number AY935499).

Obtaining sufficient 5XP WS was another difficulty as Ph. Eur. v9.0 04/2013:0450, 2013 allows flexibility over the number of birds used in the ICPI (>10 birds) and Safety tests (>20 birds). However, for five-times passaging, the monograph states that 5 birds (only) are to be used for each passage; in fact, 7 birds were used for each passage in this study to improve the chance of I-2 re-isolation between passages. Critically for us, the one big technical problem with the Ph. Eur. monograph was due to the number of birds in the fifth *in vivo* passage that limited the amount of virus available for the ICPI and Safety tests. Amplification of the I-2 titre in the 5XP WS homogenates by using embryonated eggs for a preliminary amplification phase before carrying out the ICPI and Safety tests was forbidden (Ph. Eur. Helpdesk, personal communication). To obtain the quantity of virus required, a minimum of 24 chickens would have been required for the fifth passage and to obtain sufficient material for this final challenge, the number of birds in the fourth passage would also have had to be increased substantially. This would represent a major deviation from the Ph. Eur. v9.0 04/2013:0450, 2013 that would not be permitted by the Ethics Committee of the University of Pretoria. The option of concentrating the challenge material was considered but this was not practical given the small quantity (≤5 ml) of tracheal tissue available from the birds in the challenge groups. Increasing the concentration of the challenge homogenate from a concentration of 5.1 × 10 ^6^ EID _50_ ml ^-1^ to 2.0 × 10 ^8^ EID _50_ ml ^-1^ would require the material to be concentrated approximately 40 times. It was noted that the caecal tonsils gave a significant I-2 yield and perhaps this might be of use in increasing total yields of avirulent viruses. Hopefully the Ph. Eur. monograph may be reviewed and adapted to account for the above findings (Ph. Eur. Helpdesk, personal communication) and so ease the rigours of international compliance for avirulent vaccine viruses like I-2 which are of strategic importance in pro-poor vaccination programmes. The Ph. Eur. also may need to consider both HAI and genomic quantification of viruses. Our suggestion for a review of the Ph. Eur. monograph reflects increasing knowledge and is in keeping with e.g. the suggestion to include assessments of viral shedding in vaccine safety and efficacy regulatory documents ( Cardenas-Garcia *et al.,* 2015).

I-2 was difficult to passage repeatedly *in vivo* and it induced very low ICPI values, both before and after *in vivo* passage. Sequencing data for the F _o_ site confirmed that 5XP I-2 WS had not acquired virulence here. I-2 caused no signs of overt clinical disease in a Safety eye drop challenge and overall there was no evidence of conditioning of I-2. This study confirms that Newcastle Disease vaccine virus strain I-2 is safe for use in chickens but further validation work particularly examining the reproducibility of the passage data is needed.

## Data availability

### Underlying data

Open Science Framework: Acquisition_Reversion to Virulence of I-2 NDV, https://doi.org/10.17605/OSF.IO/6Z8JM ( Domingue, 2021).

This project, among other information, contains the following underlying data:

- Quality training
- Animal husbandry and management including health observations and post-mortem analyses
- Blinding and bias reduction methods
- Preparation of brain: tracheal tissue challenge homogenates
- Five times in vivo passaging methods and virus yields (rRT-PCR and HA analyses)
- Observational scores for the safety tests for each animal
- Observational scores for ICPI tests for each animal
- F _0_ amino-acid sequence determinations

Data are available under the terms of the Creative Commons Zero "No rights reserved" data waiver (CC0 1.0 Public domain dedication).

The following data files are temporarily unavailable due to the site where these data are kept being currently under lockdown (at time of publication) owning to the COVID-19 pandemic. Once restrictions are lifted, the authors are committed to uploading the data and versioning the article so that all underlying are available to readers. In the meantime, for any clarifications about the data or analysis, readers should contact the GALVmed CEO or Chief Scientific Officer via the GALVmed website ( https://www.galvmed.org/)

- Viral titres for each animal/tissue
- Raw Ct values obtained from rRT-PCR for each run
- Raw results of the hemagglutinin inhibition test for each animal/tissue
