# Supplementary material for: Newcastle disease vaccine virus I-2 fails to acquire virulence during repeated passage in vivo
Source: Gates Open Res. 2021 Dec 23;5:76. Originally published 2021 Apr 19. [Version 3] doi: 10.12688/gatesopenres.13212.3 (PMC8595976; doi:10.12688/gatesopenres.13212.3)
Supplement: Peer review history

# Peer review: Reviewer response for version 3

**DOI:** 10.21956/gatesopenres.14732.r31591

**Published:** 2022-01-05

**Authors:** Christopher J. Morrow

**Affiliations:** 1 The University of Melbourne, Melbourne, Vic, Australia; 2 Bioproperties, Melbourne, Vic, Australia

Looking good.

Is the work clearly and accurately presented and does it cite the current literature?

Yes

If applicable, is the statistical analysis and its interpretation appropriate?

Not applicable

Are all the source data underlying the results available to ensure full reproducibility?

Partly

Is the study design appropriate and is the work technically sound?

Partly

Are the conclusions drawn adequately supported by the results?

Yes

Are sufficient details of methods and analysis provided to allow replication by others?

Partly

Reviewer Expertise:

Avian mycoplasma.

I confirm that I have read this submission and believe that I have an appropriate level of expertise to confirm that it is of an acceptable scientific standard.

# Peer review: Reviewer response for version 3

**DOI:** 10.21956/gatesopenres.14732.r31592

**Published:** 2022-01-05

**Authors:** Peter L.M. Msoffe

**Affiliations:** 1 Department of Veterinary Medicine and Public Health, Sokoine University of Agriculture, Morogoro, Tanzania

All my comments were adequately addressed by the authors. I have no further comments.

Is the work clearly and accurately presented and does it cite the current literature?

Yes

If applicable, is the statistical analysis and its interpretation appropriate?

Not applicable

Are all the source data underlying the results available to ensure full reproducibility?

Yes

Is the study design appropriate and is the work technically sound?

Yes

Are the conclusions drawn adequately supported by the results?

Yes

Are sufficient details of methods and analysis provided to allow replication by others?

Yes

Reviewer Expertise:

Poultry Health and Production

I confirm that I have read this submission and believe that I have an appropriate level of expertise to confirm that it is of an acceptable scientific standard.

# Peer review: Reviewer response for version 2

**DOI:** 10.21956/gatesopenres.14660.r31402

**Published:** 2021-11-19

**Authors:** Robyn G. Alders

**Affiliations:** 1 Global Health Programme, Chatham House, Royal Institute of International Affairs, London, UK; 2 Australian National University, Canberra, Australia; 3 Kyeema Foundation, Brisbane, Australia

Thanks to the authors for addressing most of my points. Perhaps something has gone astray with the revision process in relation to the following points:

 Keywords: change the upper case 'D' for Newcastle disease to a lower case 'd';

 Introduction: the phrase "because local registration requirements are relatively relaxed" remains problematic. This wording suggests a value judgement on the part of the authors. It would be ideal if the authors could reflect how risk assessments differ across countries and regions. While the EU regulations may be appropriate for EU countries, the risks that the EU deals with are in many cases substantially different to those faced by low-income countries. I agree with the observation by one of the co-reviewers that the OIE standards may be more appropriate given the varied circumstances under which ND needs to be controlled. The importance of national level risk assessments is covered in Young *et al.* (2012 ^1^).

 Discussion: Peter Spradbrow's title remains written as 'Dr'. Please change this to 'Professor'.

Is the work clearly and accurately presented and does it cite the current literature?

Partly

If applicable, is the statistical analysis and its interpretation appropriate?

Not applicable

Are all the source data underlying the results available to ensure full reproducibility?

Partly

Is the study design appropriate and is the work technically sound?

Yes

Are the conclusions drawn adequately supported by the results?

Yes

Are sufficient details of methods and analysis provided to allow replication by others?

Yes

Reviewer Expertise:

Newcastle disease prevention and control including ND vaccine production, quality assurance and field use.

I confirm that I have read this submission and believe that I have an appropriate level of expertise to confirm that it is of an acceptable scientific standard, however I have significant reservations, as outlined above.

## References

[1] : Controlling Newcastle Disease in Village Chickens: A Laboratory Manual (2nd edition). *Australian Centre for International Agricultural Research* .2012; Reference source

# Peer review: Reviewer response for version 2

**DOI:** 10.21956/gatesopenres.14660.r31403

**Published:** 2021-11-16

**Authors:** Christopher J. Morrow

**Affiliations:** 1 The University of Melbourne, Melbourne, Vic, Australia; 2 Bioproperties, Melbourne, Vic, Australia

The paper is clear and acceptable. Any reader can make their own decisions.

 Congratulations Shahn and Gil.

Is the work clearly and accurately presented and does it cite the current literature?

Yes

If applicable, is the statistical analysis and its interpretation appropriate?

Not applicable

Are all the source data underlying the results available to ensure full reproducibility?

Partly

Is the study design appropriate and is the work technically sound?

Partly

Are the conclusions drawn adequately supported by the results?

Yes

Are sufficient details of methods and analysis provided to allow replication by others?

Partly

Reviewer Expertise:

Avian mycoplasma.

I confirm that I have read this submission and believe that I have an appropriate level of expertise to confirm that it is of an acceptable scientific standard.

# Peer review: Reviewer response for version 1

**DOI:** 10.21956/gatesopenres.14430.r30640

**Published:** 2021-05-27

**Authors:** Peter L.M. Msoffe

**Affiliations:** 1 Department of Veterinary Medicine and Public Health, Sokoine University of Agriculture, Morogoro, Tanzania

This is an excellent study designed to generated evidence on the topical issue of ND vaccines gaining virulence after *in-vivo* passages. The fact that I-2 ND vaccine has gained acceptance and wider use in several lower and middle income countries support further the conduct of this study. It is a well designed study with very useful results and conclusions.

 However, there are a few items that need to be addressed to give the paper more strength and validity.

**Introduction section:**

1. The authors may want to modify the statement: "...the behavior of village birds is suited to the disease spread..." to more like: the behavior of the village birds make them prone or expose them to the spread of ND and other similar diseases.
2. The phrase "...of unknown genetic" is superfluous because it is not yet established if chicken genetics play any significant role the epidemiology of ND.
3. I hesitate to agree with the authors that I-2 ND vaccine is registered in some countries because of less stringent registration procedures. I think it is more because I-2 is a vaccine that responded to a real need named lack of cold chain in most lower and middle income countries! Possibly most countries that have not registered the vaccine particularly the high income ones have not done so due to lack of a pressing need since the existing thermolabile vaccine appears to be sufficient.
4. For a study that is centered on assessing the phenomenon of vaccines acquiring virulence after *in-vivo* passages, I expected this topic to be covered in the introduction and discussion sections of the manuscript including relevant citations. Absence of this information makes it difficult for an ordinary reader to see the need for such a study and hence the validity of the conclusions.
5. The authors assert that development of a global I-2 dossier will help AU-PANVAC in its pro-poor aims; however, it is not explained anywhere in the text what challenges AU-PANVAC is facing as a result of lack of global registration dossier for I-2, the statement is left hanging.

**Methods:**

- From the methodology it appears that the study design was to ensure EU acceptance rather than what the authors are describing as global acceptance. One would expect that a global acceptance would be guided by OIE or other World Organizations. 

**Results**

- It appears that the control birds were kept within the same premises with the test birds. However, it appears that no I-2 ND virus was detected in the control birds. This information seems to contradict with the established evidence that I-2 is highly transmissible within flocks. Any clarifications?
- The use of the phrase "ND challenges" when describing the ICPI results is inappropriate as it may lead to confusion to the reader whether virulent strains were used. I recommend that the authors maintain I-2 challenge throughout the text. 

Is the work clearly and accurately presented and does it cite the current literature?

Yes

If applicable, is the statistical analysis and its interpretation appropriate?

Not applicable

Are all the source data underlying the results available to ensure full reproducibility?

Yes

Is the study design appropriate and is the work technically sound?

Yes

Are the conclusions drawn adequately supported by the results?

Yes

Are sufficient details of methods and analysis provided to allow replication by others?

Yes

Reviewer Expertise:

Poultry health and production

I confirm that I have read this submission and believe that I have an appropriate level of expertise to confirm that it is of an acceptable scientific standard, however I have significant reservations, as outlined above.

# Response

**Published:** 2021-06-10

**Authors:** Gil Domingue

**Affiliations:** GALVmed, UK

1. The authors may want to modify the statement: "...the behaviour of village birds is suited to the disease spread..." to more like: the behavior of the village birds make them prone or expose them to the spread of ND and other similar diseases.

*We* a *gree.*

 2. The phrase "...of unknown genetic" is superfluous because it is not yet established if chicken genetics play any significant role the epidemiology of ND. 

*What we meant here was that there is a genetic influence upon the comparative resistance to infection; this can be manifested in bird – bird variation. We will delete this.*

 3. I hesitate to agree with the authors that I-2 ND vaccine is registered in some countries because of less stringent registration procedures, etc.

*We will delete this text.*

 4. For a study that is centered on assessing the phenomenon of vaccines acquiring virulence after *in-vivo* passages, etc

*We think that the last two paragraphs of the Introduction explained why the study was performed. We are restricted by space considerations so do not think we need to expand further.*

*5. *The authors assert that development of a global I-2 dossier will help AU-PANVAC in its pro-poor aims, etc. 

*“Boosting” the I-2 registration dossier for AU-PANVAC was just for general purposes which are now superseeded. We will delete the reference to AU-Panvac.*

*6. *From the methodology it appears that the study design was to ensure EU acceptance, etc.

*We note this comment but here we were guided by the GALVmed Technical Subcommittee (international experts) to work to OECD GLP and Ph. Eur. as international standards. There was a Galvmed policy decision to be guided more by international regulatory authorities, e.g. Ph. Eur. (tough) than OIE.*

*7. *The use of the phrase "ND challenges" , etc.

*We have noted this.*

 Additional reply:

 1. "It appears that the control birds were kept within the same premises with the test birds. etc"

*It is because I-2 virus is highly transmissible that we worked within a BSL3 standard laboratory isolation unit with separate isolation cabinets and a high efficiency ventilation system. Unique BSL3 Isolation Cabinets were used for within-test Test and Control birds. Sentinel birds were placed throughout the working areas.*

# Peer review: Reviewer response for version 1

**DOI:** 10.21956/gatesopenres.14430.r30651

**Published:** 2021-05-13

**Authors:** Christopher J. Morrow

**Affiliations:** 1 The University of Melbourne, Melbourne, Vic, Australia; 2 Bioproperties, Melbourne, Vic, Australia

This paper is a summary of an EU standard study (GLP/GCP) aimed to support registration of I-2 from the University of Queensland Master Seed. It is not a scientific study but a registration study. VICH 41 (and the derived EP and other monographs) are recognised as flawed with no positive control group to assure that an increase in virulence would be detected if it occurred during the 5 passages. (This is probably in the mistaken belief that we are reducing the number of animals used in experiments - a laudable welfare aim but if the vaccine is registered on such a flaw many more animals could suffer). Such a positive control group would assure that if the agent being tested had increased in virulence that such an increase would be detected by the challenge model being used. 

 I agree with the authors that the Ph. Eur. v9.0 04/2013:0450 is unsuitable for testing viscerotrophic NDV vaccines with its insistence that only trachael- and brain derived-inocula is used for transmission. I am not sure that the EU helpdesk advice that in vitro amplification between passaging is completely excluded is correct. Usually if direct passage without in vitro progation has been attempted and is not successful twice (as described in this paper) applicants would make a case for using in vitro passage amplification and I would have thought most (valid) ICPI testing would have used allantoic fluid and it may not have been validated for the final passage material being assessed and therefore brain and tracheal homogenate would need extra controls in the ICPI test. At least in the final passage before the safety testing in vitro passage may be needed).

 The statement that "Currently only one serotype of ND virus is recognised" is tautological as haemagglutinating viral isolates are identified as NDV by their serological characterization by sera raised against a NDV type strain raised antibody.  

 The stated aim of the project to generate local manufacture of inexpensive vaccine is now an anachronism with regulation creep imposing first world quality standards on vaccines in nearly all countries. The need for SPF eggs for vaccine manufacture  and GMP standards are nearly universal and an expense. The need to protect chickens from viral and other contaminates is hard to argue against (having suffered through REV, EDS-76, ALV, CAV over the years). The materials and methods do not state whether SPF eggs were used for the initial propagation of the material studied.

 It is not clear what SPF definition has been used for this work.

Is the work clearly and accurately presented and does it cite the current literature?

Yes

If applicable, is the statistical analysis and its interpretation appropriate?

Not applicable

Are all the source data underlying the results available to ensure full reproducibility?

Partly

Is the study design appropriate and is the work technically sound?

Partly

Are the conclusions drawn adequately supported by the results?

Yes

Are sufficient details of methods and analysis provided to allow replication by others?

Partly

Reviewer Expertise:

Avian mycoplasma.

I confirm that I have read this submission and believe that I have an appropriate level of expertise to confirm that it is of an acceptable scientific standard, however I have significant reservations, as outlined above.

# Response

**Published:** 2021-06-10

**Authors:** Gil Domingue

**Affiliations:** GALVmed, UK

1. This paper is a summary of an EU standard study (GLP/GCP) aimed to support registration of I-2 from the University of Queensland Master Seed. It is not a scientific study but a registration study. VICH 41 (and the derived EP and other monographs) are recognised as flawed with no positive control group to assure that an increase in virulence would be detected if it occurred during the 5 passages. ...etc.

*We agree this to be the case but the intention was to follow the Ph. Eur. as an accepted international standard for regulatory purposes. *A *n early Galvmed policy decision handed down by GALVmed's independent Technical Subcommittee (international experts) was to be guided more by international regulatory authorities than by OIE.. Therefore the Ph. Eur. (more demanding) became the international standard for us. As a result, the study design is acceptable from a regulatory point of view but lacks positive controls. However we never detected any "conditioning" that enhanced the virulence of I-2 ND virus.*

*2. *I agree with the authors that the Ph. Eur. v9.0 04/2013:0450 is unsuitable for testing viscerotrophic NDV vaccines with its insistence that only trachael- and brain derived-inocula is used for transmission. I am not sure that the EU helpdesk advice that in vitro amplification between passaging is completely excluded is correct etc.

*The Ph. Eur. Helpdesk* *confirmed in writing (in our confidential records) that using embryonated SPF eggs for a preliminary amplification phase before carrying out the ICPI and safety tests was forbidden; this after being informed that we needed to do a second series. There was no reason to proceed to a third series when we might have got permission to use in vitro passage amplification.*

*3. *The statement that "Currently only one serotype of ND virus is recognised" is tautological etc.

*We accept this and will delete the statement*.

 4. The materials and methods do not state whether SPF eggs were used for the initial propagation of the material studied. It is not clear what SPF definition has been used for this work etc.

*SPF eggs were used throughout including for MS inoculation. We will revise the text to make this clearer.*

*Our Specific Pathogen Free (SPF) Eggs were fertile chicken eggs produced from known SPF parent flocks established and maintained as per the recommendations of a committee appointed in 1974 by the International Association of Biological Standardization (IABS) Geneva, chaired by Dr. R. Luginbhul. We do not feel our manuscript would gain from including this detail.*

# Peer review: Reviewer response for version 1

**DOI:** 10.21956/gatesopenres.14430.r30601

**Published:** 2021-05-04

**Authors:** Robyn G. Alders

**Affiliations:** 1 Global Health Programme, Chatham House, Royal Institute of International Affairs, London, UK; 2 Australian National University, Canberra, Australia; 3 Kyeema Foundation, Brisbane, Australia

This study builds on previous *in vitro* and *in vivo* research by investigating whether the naturally attenuated, thermotolerant Newcastle disease vaccine virus I-2 could acquire virulence after five *in vivo* passages through SPF chickens. This is an important contribution to the literature.

 The article can be improved by the addition of key information and some re-phasing of statements as indicated below.

**Specific comments:**

**Abstract & Keywords:**

- Please correct the writing of Newcastle disease so that it is in line with OIE standards, i.e. ‘Newcastle’ is written with an upper case ‘N’ whereas ‘disease’ starts with a lower case ‘d’.

**Abstract:**

- Last sentence: please reword to indicate that this current study provides additional and definitive evidence that the I-2 ND strain is safe to use. Significant studies have been done previously into the safety of the I-2 ND strain, including multiple passages in embryonating eggs. It has found not to cause clinical signs at 100x the standard dose in chickens. It causes no clinical signs when administered to Day Old Chicks.

**Introduction:**

- Please revise the statement “Unfortunately the behaviour of village birds is suited to disease spread” as this is incomplete and somewhat misleading. In terms of disease transmission, reproductive rates of pathogens are higher in intensively raised poultry and the frequent movement of birds and fomites along poultry value chains facilitates disease spread. That the village should be considered the epidemiological unit for village poultry was clearly understood during the HPAI H5N1 pandemic. It was the intensive commercial poultry industry that disproportionately facilitated the wide spread of the H5N1 subtype as reported in multiple papers.
- Please revise the statement “ND I-2 vaccine use has also been allowed in these low-middle income countries because local registration requirements are relatively relaxed, but the vaccine has not been registered in those countries where registration requirements are more demanding.” In the countries where the I-2 ND is approved for use, it was usually the first vaccine to go through a registration process and so contributed to building regulatory capacity in these countries. Information dossiers were prepared in each case.
- Please revise the statement “Indeed one of the obstacles to the wider use of the I-2 vaccine has been that no comprehensive dossier has been compiled on the vaccine” as this is incorrect. Detailed information dossiers have been prepared for the I-2 ND vaccine produced in a number of countries. The dossiers included *in vitro* and *in vivo* safety test results. The findings presented in this paper provides further evidence that the I-2 ND vaccine is safe for use.

**Discussion:**

- Please change “Dr Spradbrow” to “Professor Spradbrow”.

Is the work clearly and accurately presented and does it cite the current literature?

Partly

If applicable, is the statistical analysis and its interpretation appropriate?

Not applicable

Are all the source data underlying the results available to ensure full reproducibility?

Partly

Is the study design appropriate and is the work technically sound?

Yes

Are the conclusions drawn adequately supported by the results?

Yes

Are sufficient details of methods and analysis provided to allow replication by others?

Yes

Reviewer Expertise:

Newcastle disease prevention and control including ND vaccine production, quality assurance and field use.

I confirm that I have read this submission and believe that I have an appropriate level of expertise to confirm that it is of an acceptable scientific standard, however I have significant reservations, as outlined above.

# Response

**Published:** 2021-06-11

**Authors:** Gil Domingue

**Affiliations:** GALVmed, UK

1. Please correct the writing of Newcastle disease so that it is in line with OIE standards, etc.

*Accepted.*

 2. Last sentence: please reword to indicate that this current study provides additional and definitive evidence that the I-2 ND strain is safe to use.

*We do not find our Abstract final sentence is very different from that suggested. Also we are restricted to 300 words. We think the point is that our study was conducted to be definitive from an international regulatory perspective.*

*3. *Please revise the statement “Unfortunately the behaviour of village birds is suited to disease spread” etc.

*Accepted; Dr Msoffe, one of our reviewers and lead author on the paper we reference here, has suggested we rewrite this as “the behaviour of the village birds make them prone to or exposes them to the spread of ND and other similar diseases”. We will revise this.*

 4. Please revise the statement “ND I-2 vaccine use has also been allowed in these low-middle income countries because local registration requirements are relatively relaxed, etc.

*GALVmed was advised by its independent Technical Subcommitee comprised of international experts. who informed us that to their knowledge at the time, "no comprehensive dossier of an international regulatory standard had been compiled on the vaccine". We can revise the text.*

 5. Discussion.Please change “Dr Spradbrow” to “Professor Spradbrow”.

*Accepted.*
